# Current Potential Therapeutic Approaches against SARS-CoV-2: A Review

**DOI:** 10.3390/biomedicines9111620

**Published:** 2021-11-04

**Authors:** Dharmendra Kumar Yadav, Desh Deepak Singh, Ihn Han, Yogesh Kumar, Eun-Ha Choi

**Affiliations:** 1 Gachon Institute of Pharmaceutical Science and Department of Pharmacy, College of Pharmacy, Gachon University, Hambakmoeiro 191, Yeonsu-gu, Incheon 21924, Korea; 2 Amity Institute of Biotechnology, Amity University Rajasthan, Jaipur 303002, India; ddsbms@gmail.com; 3 Plasma Bioscience Research Center, Applied Plasma Medicine Center, Department of Electrical & Biological Physics, Kwangwoon University, Seoul 01897, Korea; difflvin@hotmail.com; 4 Department of General, Visceral and Thoracic Surgery, University Medical Center Hamburg-Eppendorf, Martinistrasse 52/Gebäude N27, 20246 Hamburg, Germany; kumar.yogesh601@gmail.com

**Keywords:** SARS-CoV-2, combination therapy, virus-based therapy, host-based therapy, SARS-CoV-2 cell entry inhibitors

## Abstract

The ongoing SARS-CoV-2 pandemic is a serious threat to public health worldwide and, to date, no effective treatment is available. Thus, we herein review the pharmaceutical approaches to SARS-CoV-2 infection treatment. Numerous candidate medicines that can prevent SARS-CoV-2 infection and replication have been proposed. These medicines include inhibitors of serine protease TMPRSS2 and angiotensin converting enzyme 2 (ACE2). The S protein of SARS-CoV-2 binds to the receptor in host cells. ACE2 inhibitors block TMPRSS2 and S protein priming, thus preventing SARS-CoV-2 entry to host cells. Moreover, antiviral medicines (including the nucleotide analogue remdesivir, the HIV protease inhibitors lopinavir and ritonavir, and wide-spectrum antiviral antibiotics arbidol and favipiravir) have been shown to reduce the dissemination of SARS-CoV-2 as well as morbidity and mortality associated with COVID-19.

## 1. Introduction

Coronaviruses contain positive-sense, single-stranded RNA, with a genome size ranging from 26–32 kb, and five structural proteins, and are classified into four categories: alpha, beta, gamma, and delta [1,2]. Human coronaviruses are alpha and beta coronaviruses which can cause respiratory and gastrointestinal tract infections [2]. The severe acute respiratory syndrome (SARS) outbreak between November 2002 and July 2003 (nine months) resulted in more than 8000 total cases and 774 deaths, with a fatality rate of 9.6% [3]. Middle East respiratory syndrome (MERS) was reported in 2012 resulting in more than 2400 cases and 858 deaths, with a fatality rate of 34.4%. Subsequently, in late December 2019, an unspecified case of pneumonia was reported in Wuhan, Hubei Province, the People’s Republic of China [1,2,3]. COVID-19 is the official name given by the WHO to the disease caused by SARS-CoV-2 infection. It has since been observed that the virus could spread from human to human [4]. Its incubation period is 2 to 14 days with various clinical presentations: asymptomatic, mild to severe illness, and mortality [5]. Symptoms include fever, cough, difficulty breathing, malaise and fatigue, gastrointestinal symptoms (decreased appetite, vomiting, watery diarrhea, and dehydration), loss of taste and smell, sore throat, rhinorrhoea, severe pneumonia, and acute respiratory distress, which can lead to multiple organ failure and death. The SARS-CoV-2 virus is mainly spread via airborne/aerosol particles; the virus has been observed to remain viable and infective for over 3 h in the air [6,7]. SARS-CoV-2 infection is a highly communicable disease, and this pandemic has been designated a world public health emergency by the World Health Organization (WHO) [7]. However, SARS-CoV-2 has many potential natural, intermediate, and final hosts, as do other viruses; thus, major problems in the prevention and diagnosis of viral infection are raised [8]. In this paper we discuss the genetic structure of SARS-CoV-2 and its mechanism of pathogenesis. We include consideration of the phylogenetic analysis of the SARS-CoV-2 genome, multiple sequence alignment analysis, and therapeutic approaches to SAR-Co-V-2 infection.

## 2. SARS-CoV-2 Genetic Structure and Pathogenic Mechanism

**Figure 1:**
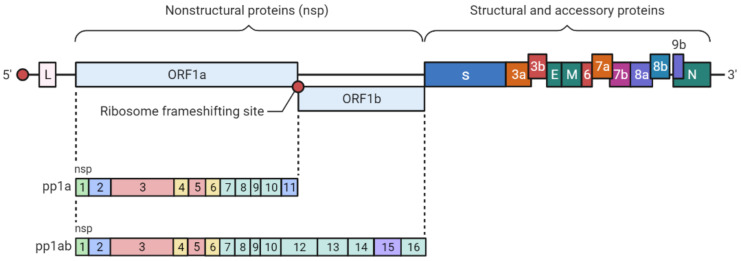
Genome structure of SARS-CoV-2. Figure was created by using BioRender (https://biorender.com, accessed on 15 September 2021).

**Figure 2:**
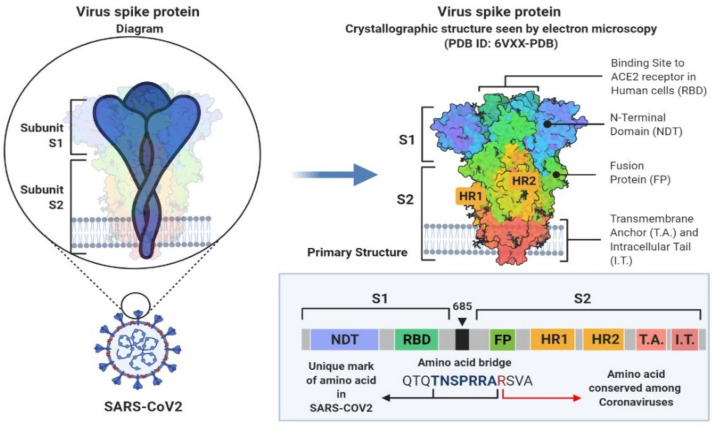
Crystallographic structure SARS-CoV-2. Figure was created using by BioRender (https://biorender.com, accessed on 15 September 2021).

The SARS-CoV-2 genome codes for more than 20 distinct proteins. At least four structural proteins are present in coronaviruses, namely spike (S), envelope (E), membrane (M), and nucleocapsid (N) proteins (Figure 1). S proteins, which are involved in host attachment and virus-cell membrane fusion, determine the host range for viral infection (Figure 2) [9].

The SARS-CoV-2 main protease (Mpro) is recognised as one of the most essential viral proteins. SARS-CoV-2 Mpro is more than 96% similar to SARS-CoV Mpro. During viral translation, SARS-CoV-2 Mpro cleaves 11 polyproteins to polypeptides that are required for transcription and replication [10]. Some of the candidate drugs that can prevent SARS-CoV-2 viral replication target Mpro, such as remdesivir, griffithsin, nafamostat, disulfiram, lopinavir/ritonavir, nelfinavir, danoprevir and favipiravir [11].

## 3. Phylogenetic Analysis of SARS-CoV-2 Genome

**Figure 3:**
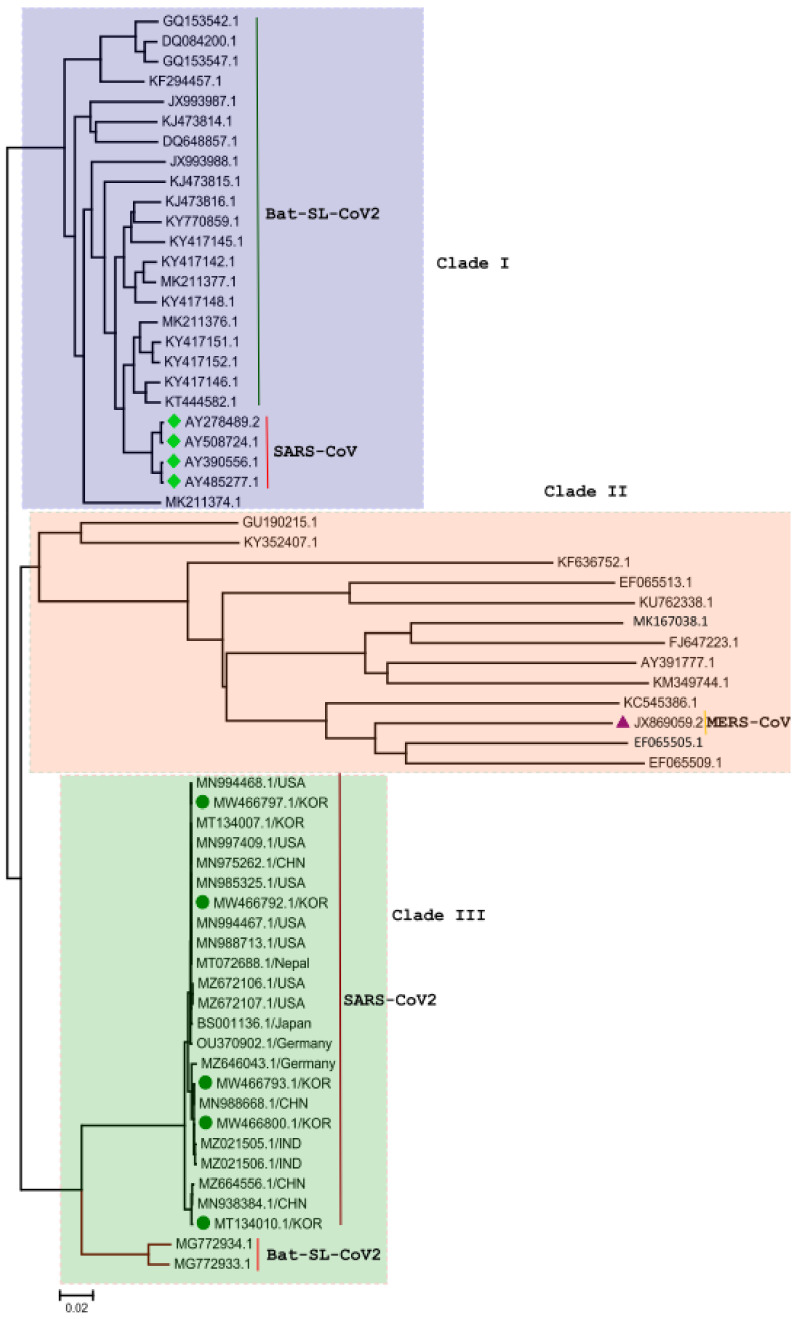
The phylogenetic tree was generated using the latest complete genome sequences of different neighbors, MERS-CoV, SARS-CoV, and Bat-SL-CoV. The tree is divided into three major clades according to the grouping of clusters: Clade I: Bat-SL-CoV-2 and SARS-CoV viruses showing a close evolutionary relationship with each other. Clade II: Human and bat coronaviruses, including MERS-CoV. Clade III: All of the SARS-CoV-2 genomes isolated from humans—it was observed that these genomes show a close evolutionary relationship with Bat-SL-CoV-2.

**Figure 4:**
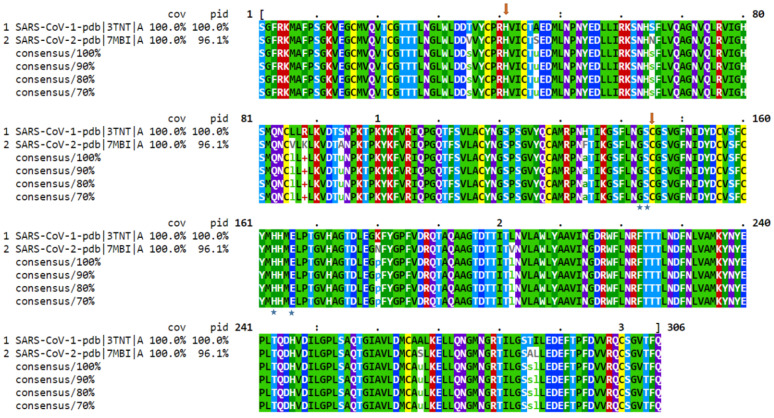
Multiple sequence alignment analysis of the amino acid sequence of SARS-CoV-1 and SARS-CoV-2 Mpro. Amino acids marked underneath with an arrow represent catalytic residues; residues marked underneath with * represent substrate-binding residues of various subsites.

A sequence alignment and phylogenetic analysis of SARS-CoV-2 genome is shown in Figure 3. The phylogenetic tree is primarily divided into three clades [12]. Clade I consist of SARS-CoV and Bat-SL-CoV genomes which share a sequence identity ranging from 88% to 99%. Clade II consist of 13 complete genomes of coronavirus and MERS-CoV genomes which share a sequence identity from 78% to 89%. Clade III consist of 23 SARS-CoV-2 and Bat-SL-CoV complete genomes which share a sequence identity ranging from 89% to 100%; the SARS-CoV-2 genomes isolated from human samples show a sequence identity ranging from 98% to 100% [13]. A particularly interesting observation from the analysis was that there is no major divergence in the SARS-CoV-2 genome sequence of different SARS-CoV-2 virus genomes isolated from different countries, as shown in Figure 3. The sequence alignment of the SARS-CoV-1 (Bat, PDB ID: 3TNT) and the SARS-CoV-2 (human, PDB ID: 7MBI) main proteases reveals that the amino acid sequence is conserved with a sequence identity of 96%; differences between these genomes are shown in Figure 4 at specific positions [13,14].

## 4. Therapeutic Approaches to SAR-COV-2 Infection

**Figure 5:**
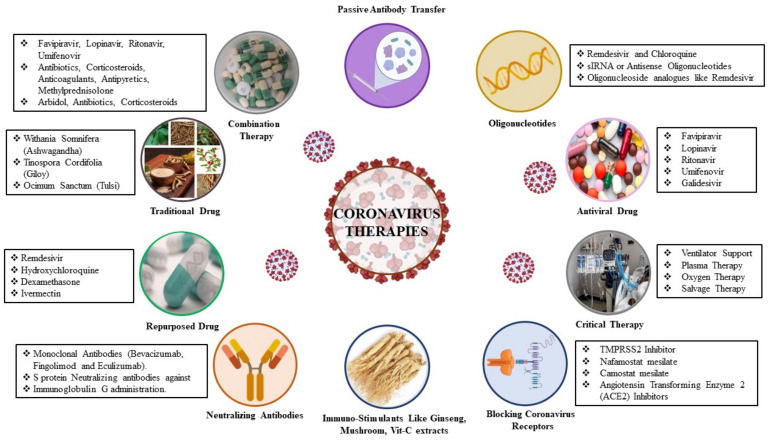
Therapeutic approaches to SARS-CoV-2 infection.

To identify therapeutic agents that are effective against SARS-CoV-2 infection, extensive research on the structure and pathogenesis of COVID-19 is in progress [15]. Therapeutic approaches to COVID-19 can be categorized into virus-based therapy and host-based therapy, as shown in Figure 5.

### 4.1. Virus-Based Therapy

Viral nucleic acids consist of nucleosides and nucleotides. Drugs capable of attacking nucleotides, nucleosides, or viral nucleic acids can affect the activity of a broad range of coronaviruses and other viruses, as shown in Table 1 [16]. Possible targets for antiviral therapy include major enzymes and proteins involved in SARS-CoV-2 viral replication. The PLpro enzymes and papain-like protease of SARS-CoV and MERS-CoV have been shown to exert proteolytic, deubiquitylating, and deISGylating activities. Studies have shown that lopinavir-ritonavir is the most potent protease inhibitor as shown in Table 1 [17]. The main SARS-CoV-2 immunogen antigen is the Spike glycoprotein with membrane anchor, which plays an important role in the interaction between host cells and viruses. Studies have shown that certain monoclonal antibodies can target the receptor binding domain (RBD) subunit epitopes and inhibit viral cell receptor binding, whereas other monoclonal antibodies bind to the S2 subunit and disrupt viral cell fusion [18]. A study using the CR3022 neutralising antibody of SARS-CoV shown in Table 1 [19]. Earlier trials also showed that adoptive transfer of plasma containing anti-MERS-COV-S antibodies had the ability to prevent infection and accelerate viral clearance.

### 4.2. Host-Based Therapy

Viral entry of SARS-CoV-2 depends on the priming of its spike protein and on transmembrane protease 2 (TMPRSS2). Further studies have shown that camostat mesylate, a serine protease inhibitor, can block TMPRSS2 activity and is thus considered as a therapeutic candidate as shown in Table 2 [33]. Other research indicates a pH- and receptor-dependent endocytosis when coronavirus is introduced into the host cell. AP-2-associated protein kinase 1 (AAK1), a host kinase, controls clathrin-mediated endocytosis [34]. Since the virus structure is now established, various inhibitors have been tested in cell-based systems for their ability to prevent viral entry and replication within the host body, as shown in Table 2 [35]. These include spike (S) protein inhibitors, S-cleavage inhibitors, helicase and protease inhibitors, fusion core blockers, HCB monoclonal antibodies, RBD–ACE2 blockers, antiviral peptides, siRNAs, and antifreeze eutralizati antibodies [35,36]. The following section concentrates on the possible therapeutic treatment options based on our limited knowledge of SARS-CoV-2.

#### 4.2.1. Neutralizing Antibodies

**Figure 6:**
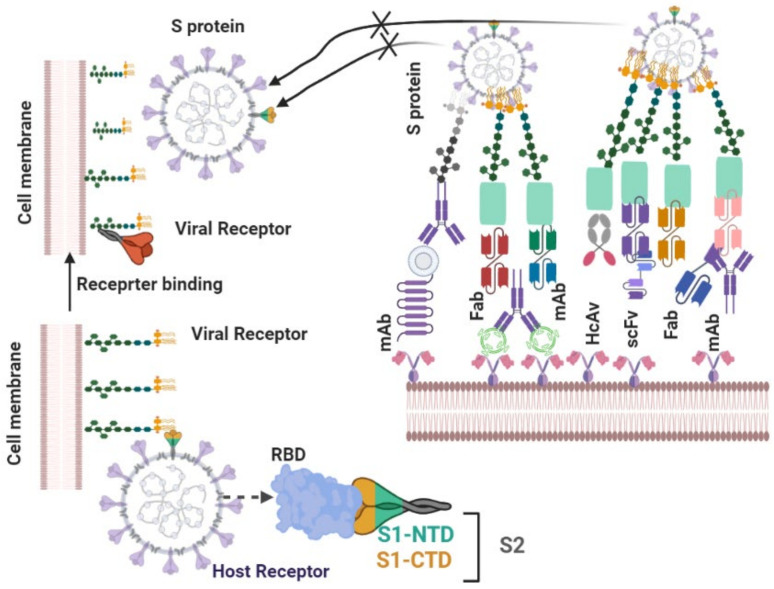
Schematic of binding mechanism of SARS-CoV-2 spike protein to the receptor.

In general, coronavirus infection begins with the entry of the viral S protein, which binds to the cell surface. This S protein fuses with the cell membrane and facilitates the syncytial development and transmission of viral nucleocapsids into the cell for further replication [35]. Studies have shown that neutralization of the S protein RBD of SARS-CoV [36] and MERS-CoV [38,39,40] by antibodies can be effective against these diseases. Neutralisation of antigens can be highly useful in COVID-19 treatment, given that the S protein RBD sequence of SARS-CoV-2 is similar to those of SARS-CoV and MERS-CoV [37]. Critical COVID-19 patients are currently treated with immunoglobulin G [35,36]. FcR plays a role in inflammation in the lung; therefore, inflammation in COVID-19 can be reduced by blocking FcR activation. Thus, intravenous administration of immunoglobulins can be effective in the treatment of pulmonary inflammation, as shown in Table 3 and Figure 6 [41].

#### 4.2.2. Antiviral Proteases

Lopinavir has been suggested as a treatment choice for persistent COVID-19 infection [41], as it inhibits the activity of coronavirus proteases. In addition, an inhibitor of vinyl sulfone protease has recently been proposed for treating patients with COVID-19 and can potentially be used widely as an anti-coronaviral agent [42]. Recently, the elucidation of the SARS-CoV-2 Mpro structure has brought about a breakthrough in antiviral studies, and numerous new drug candidates have been developed since. The Deep Docking platform, a virtual screening platform for structural proteins, containing approximately 1.2 billion molecules and 1000 suspected SARS-CoV-2 Mpro proteins, was recently established [42].

#### 4.2.3. Combination Therapy

Combinations of wide-spectrum antivirals, such as lopinavir-ritonavir, neuraminidase inhibitors, peptides (EK1), and RNA synthesis inhibitors, have been tested as anti-viral treatments. The combination of antiviral medicines, however, is controversial [43]. Patients with COVID-19 have been treated with oseltamivir, lopinavir, ritonavir, and ganciclovir [44]. Additional medications have included antibiotics (e.g., cephalosporins, quinolones, carbapenems, methicillin-resistant *Staphylococcus aureus* tigecycline, linezolid, moxifloxacin, levofloxacin, azithromycin, or amoxicillin), antifungals, and corticosteroids (prednisolone or dexamethasone) [45].

Traditional Indian medicine, which includes Ayurveda, Siddha, Unani and Yoga, naturopathy, and homeopathy, is one of the oldest therapies in human history and has been used to treat various diseases [46]. However, numerous other drugs for COVID-19 patients are still being tested. Teicoplanin is a staphylococcus glycopeptide [45] recommended for the treatment of complex COVID-19 cases and *S. aureus* infections [45], and has been shown to be effective in treating complications in these patients. In a clinical trial, danoprevir, a viral protease inhibitor, led to substantial improvement in patients with COVID-19 [47]. A summary of the development of therapeutics against COVID-19 is shown in Figure 5.

#### 4.2.4. SARS-CoV-2 Cell Entry Inhibitors

##### Inhibitors of TMPRSS2 Serine Protease

Transmembrane protease serine subfamily 2 (TMPRSS2), an airway and alveolar cell serine protease, is regulated by the cleavage and activation of the SARS-CoV spike protein (S protein), which is necessary for membrane fusion and host cell entry [48]. Camostat mesylate is a clinically proven serine defence inhibitor that partially prevents infection of HeLa cells expressing the ACE2 and TMPRSS2 by HCoV-NL63 and SARS-CoV-2 [49].

##### Nafamostat Mesylate

Nafamostat mesylate (FUT-175; CAS number: 81525-10-2) is an artificial serine protease inhibitor clinically approved in Japan for the treatment of acute pancreatitis, intravascular coagulation dissemination, and extracorporeal circulation antioxidation [49,50]. Through screening of approximately 1100 drugs for those that can inhibit the fusion of TMPRSS2, the FDA determined that nafamostat mesylate inhibited the fusion of TMPRSS2-expressing Calu-3 host cells with the MERS-CoV S protein [49,50].

##### Camostat Mesylate (Foipan™)

In a clinical trial, Foipan™ [(*N*,*N*-dimethylcarbamoylmethyl (4-(4-guanidinobenzoyloxy)-phenylacetate)) methanesulfate] [51], also known as camostat mesylate (NI-03; CAS number: 59721-28-7), was administered at a dose of 200 mg three times a day for 2 weeks to 95 patients to investigate its effect against dyspepsia associated with non-alcoholic mild pancreatic disease; the drug showed mild, but no serious, adverse effects, which indicated that camostat mesylate was a well-tolerated medicine [51,52].

##### Angiotensin Transforming Enzyme 2 (ACE2) Inhibitors and Antimalarial Drugs

SARS-CoV and corresponding coronaviruses interact directly with the host cell exoprotease and metallocarboxypeptidase (ACE2, S) via their S proteins, with angiotensin converting enzyme 2 (ACE2) catalysing the conversion of angiotensin I into nonapeptide angiotensin and angiotensin II into angiotensin 1–7 [53,54]. Unfortunately, the ACE inhibitors commonly used against hypertension and chronic heart failure cannot inhibit ACE2 [53,54]; however, other drugs and compounds have been shown to inhibit ACE2 [53].

##### Cepharanthine/Selamectin/Mefloquine Hydrochloride

The triple combination of mefloquine hydrochloride (Lariam™, used for the prophylaxis and treatment of malaria), cepharanthine (an anti-inflammatory alkaloid from *Stephania cepharantha* Hayata; and selamectin (an avermectin isolated from *Streptomyces avermitilis* and used as an anthelminthic and parasiticide drug in veterinary medicine; CAS number. 220119-17-5) [55,56] have recently been shown to inhibit the infection of simian Vero E6 cells with pangolin coronavirus GX_P2V/2017/Guangxi (GX_P2V), whose S protein shares 92.2% amino acid identity with that of SARS-CoV-2 [55,56].

##### Chloroquine Phosphate and Hydroxychloroquine

Chloroquine has been used for decades in malaria prophylaxis and for the treatment of chronic Q fever and autoimmune illnesses [57]. Hydroxychloroquine is transformed into chloroquine in vivo. These drugs have recently been shown as possible broad-spectrum antiviral medications [58]. Chloroquine phosphate inhibits ACE2 terminal phosphorylation and increases pH, both of which constitute the antiviral mechanisms of chloroquine and hydroxychloroquine [57,58].

#### 4.2.5. Inhibitors of the Replication, Membrane Fusion, and Assembly of SARS-CoV-2

##### Lopinavir/Ritonavir (Kaletra™)

Lopinavir (ABT-378) was developed in 1998 to overcome HIV resistance to the protease inhibitor ritonavir (ABT-538), which is caused by a valine mutation at position 82 (Val 82) on an active site of HIV protease in response to ritonavir treatment [38]. As the metabolism of lopinavir is strongly inhibited by ritonavir, lopinavir has been administered at oral dosages exceeding ritonavir by >50-fold in rat, dog, and monkey-plasma for an in vitro EC50 for lopinavir after 8 h [39]. Lopinavir-ritonavir treatment was associated with inconsistencies in clinical improvement, but showed comparable mortality with the standard-care treatment after 28 days of treatment [38,39].

##### Favipiravir

Favipiravir (Avigan™; T-705) is an oral pyrazinecarboxamide derivative developed by Toyama Chemical, China. It is a potent, selective inhibitor of the RNA-dependent RNA polymerase (RdRp) of RNA viruses, inducing lethal RNA transversion mutations and consequently, producing a nonviable virus phenotype [40]. Favipiravir is effective against various RNA viruses, including influenza A virus, flavivirus, alphavirus, filovirus, bunyavirus, arenavirus, norovirus, West Nile virus, yellow fever virus, foot-and-mouth disease viruses, Ebola virus, and Lassa virus [59].

##### Umifenovir

Umifenovir(ethyl-6-bromo-4-[(dimethylamino)methyl]-5-hydroxy-1-methyl-2 [(phenylthio) methyl]-indole-3-carboxylate hydrochloride monohydrate; CAS number: 131707-25-0) is a small indole-derivate molecule manufactured by JSC Pharmstandard, Russia [59]. Umifenovir prevents viral host cell entry by inhibiting fusion of the viral envelope and host cell cytoplasmic membrane through inhibition of clathrin-mediated endocytosis, thereby preventing virus infection [60].

##### Inhibitors of SARS-CoV-2 3CLpro Protease

3CLpro (also known as Mpro) is the major betacoronavirus protease necessary for viral RNA translation in polyprotein synthesis [10,30]. Recently, the x-ray structures and α-ketoamide complex of SARS-CoV-2 3CLpro have been reported [10,30,61]. Two compound pulmonary tropism pyridine-containing α-ketoamides, called 13a and 13b, exerted pharmacokinetic properties at sufficient concentration in the lungs and bronchial-alveolar liquid lavage of mice within 4 to 24 h of administration [47]. N3, formerly known as Michael acceptor inhibitor, was developed using a computer-aided method. N3 can specifically inhibit the Mpro of multiple coronaviruses, including SARS-CoV and MERS-CoV, and has shown potent antiviral activity against infectious bronchitis virus in an animal model [51,52,61]. N3 was shown to form a covalent bond with SARS-CoV-2 3CLpro, thereby acting as an irreversible inhibitor of SARS-CoV and MERS-CoV 3CLpro [51,52].

##### SARS-CoV-2 Mpro Inhibitor

**Figure 7:**
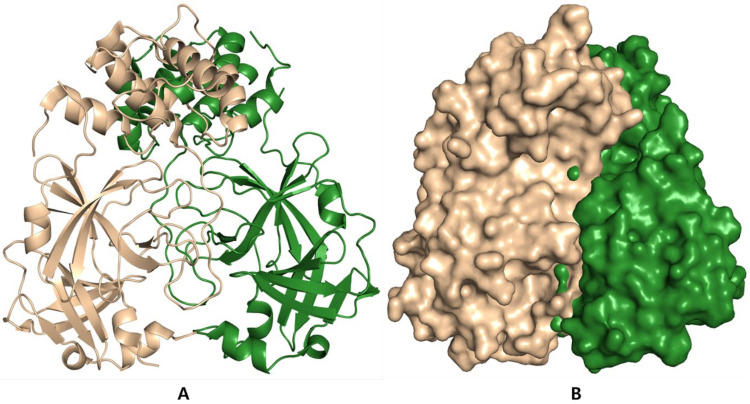
(**A**) Structural features of the main protease of SARS-CoV-2 dimer. A SARS-CoV-2 main protease. (**B**) Sphere representation of main protease monomer, in the active site groove.

The SARS-CoV-2 Mpro structure in the apo form and α-ketoamide bound with the protein form a dimer crystallographic composed of two monomers of identical conformations [62]. Each protomer is made up of three domains, as shown in Figure 7A,B, Domain I (residues 8–101) and II (residues 102–184) interface to form the active site of the protein, where α-ketoamide derivative bound complex and composed of a Cys145-His41 dyad with an antiparallel β-barrel structure. Domain III (residues 201–303) contains five α-helices, connected to domain II by a long loop region (residues 185–200), with the substrate-binding site located in a cleft between domain I and domain II. It has shown several highly conserved interactions and forms a catalytic bond with the His41 and Cys145 and adjacent residues in the substrate binding cleft such as Gly143 and Ser144 dyad. SARS-CoV uses a chymotrypsin-like protease (3CLpro) and a papain-like protease (PLpro) to process and cleave its long polyprotein precursor into individually functional non-structural proteins [63,64].

**Figure 8:**
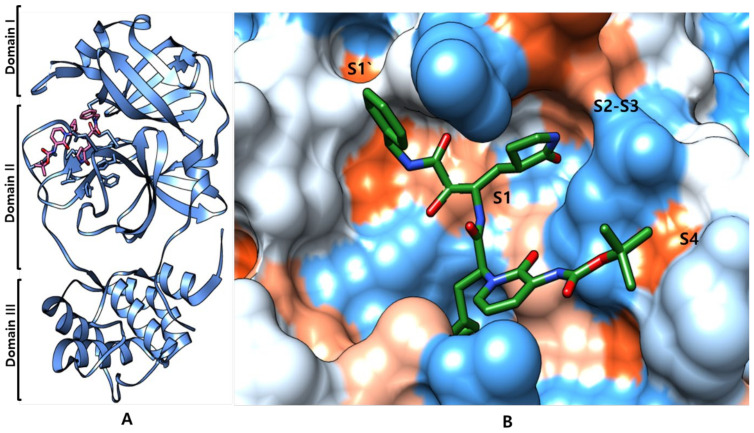
(**A**) Structural features of the main protease of SARS-CoV-2 monomer and consists of three domains. A linker joins domain II to domain III, which is critical for the dimerization of protein. (**B**) Different S1′, S1, S2, S3 and S4 subsites groups in the substrate-binding subsites of SARS-CoV-2 M^Pro^.

In the active site region of SARS-CoV-2 Mpro, the S1 residues contributed by Cys145, Gly143, and Ser144 also serve as the oxyanion hole. Bulky Gln189 and Pro168 residues form the S4 site, the S1 residue is His163, while Glu166 and Gln189 are located at the S2 position (Figure 8A). The Mpro recognizes and binds specific residues at each subsite of the peptide substrate to determine the initiation of proteolysis and the production of non-structural proteins for the formation of the replication-transcription complex. Crystal structures of Mpro have revealed that the most variable regions are the helical domain III and surface loops, and that the substrate-binding pocket (located in a cleft between domain I and domain II) is highly conserved among Mpro in all coronaviruses; this suggests that antiviral inhibitors targeting this pocket should have wide-spectrum activity against coronaviruses, as shown in Figure 8B. A previous study proposed that Mpro has a substrate-recognition pocket that is highly conserved among all coronaviruses and that this pocket could serve as a drug target for the design of broad-spectrum inhibitors. The recent discovery of new coronaviruses, and the accumulation of structural data for Mpro from coronaviruses of various species, has provided the opportunity to further examine this hypothesis.

## 5. Nanomaterials Approach for Anti-Coronavirus

Nanomaterials, such as silver colloid, titanium dioxide, and diphyllin nanoparticles, are considered promising antiviral agents and drug-delivery platforms for the effective management of coronavirus infection (Table 4, Table 5 and Table 6) [65,66,67,68,69,70,71,72]. Small interfering RNAs (siRNAs) are highly efficient in reducing the replication of RNA viruses, such as coronaviruses. The efficacy of siRNA-based treatments strictly depends on specific targeting of the viral sequence of interest and targeted cellular delivery of therapeutic siRNA [73]. In this context, nontoxic, biocompatible nanocarriers composed of polymers, lipids, polymer/lipid hybrid nanoparticles, nanohydrogels, silica, dendrimers, iron oxide nanoparticles, or gold nanoparticles are considered as promising siRNA-delivery platforms [74]. These nanocarriers can improve siRNA stability by preventing enzymatic degradation. For inhalable antiviral siRNA loading and aerosol-based delivery of antiviral siRNA in the lungs, polymer/lipid nanocarriers have shown promising outcomes [73,74].

Antiviral nanoparticles have been used as potential immunostimulatory agents for vaccine development. For example, gold nanoparticles conjugated with swine transmissible gastroenteritis virus have been used to activate macrophages, induce interferon production, and increase anti-coronavirus neutralizing antibody levels in vaccinated animals [75]. Similarly, conjugates of ribonucleic acid and ferritin-based nanoparticles have been used as molecular chaperons to develop a vaccine against MERS-CoV [74,75]. The vaccine has been shown to induce a strong T cell response and promote interferon production. Currently, nanotechnology is playing an increasingly important role in antiviral therapy for coronaviruses [73,75]. Nanomaterials have been developed specifically to improve the delivery of biotherapeutics across physiological barriers [73,74,75]. A broad range of potential nanodevices, such as nanosensors, nano-based vaccines, and smart nanomedicines, offer great hope for combating current and future mutated versions of coronaviruses [72].

**Table 4:** List of Nanomaterial’s approach against coronavirus.

Nanomaterial	Chitosan Nanospheres	CQDs^c)^ Nanocrystals4–	Gold Nanorods	PLGA^g)^ Hollow Nanoparticles	TiO_2_ Nanoparticles	Ref
**Average size**	10 nm–10 µm	9 nm	18–54 nm	114 nm	Not reported	[65,66,67,68,69,70,71,72,73,74,75]
**Shape**	Spherical	Spherical	Rod	Spherical	Predominantly spherical	
**Strategy**	Genipin-crosslinked chitosan	Boronic acid-functionalized CQDs (Carbon quantum dots)	Gold nanorods-based peptides	Viral antigens and STING agonists-loaded hollow nanoparticles	TNPs^i)^-coated glass coverslips UVC radiation	
**Coronavirus**	HCoV NL63 (Human coronavirus NL63)	HCoV-229E-Luc	MERS-CoV	MERS-CoV	HCoV NL63^a)^	
**Potential application**	Adsorbents	Antiviral drugs	Antiviral drugs	Vaccine	Self-cleaning surfaces	

**Table 5:** Nanomaterial’s approach against SARS-CoV-2.

Nanomaterial	Iron Oxide Nanoparticles	Poly Silica Nanoparticles	Gold Nanoparticles	Nano-Sized Formazans	Polylactic-Co-Glycolic Acid (PLGA)-Nanoparticles	Silver Nanoparticles	Ref.
**Size**	N/r^a^	210 ± 40 nm	N/r^a^	23.75 ± 7.16 nm	N/r^a^		[65,66,67,68,69,70,71,72,73,74,75,76]
**Strategy**	Nano-mineral structure of Fe_2_O_3_ and Fe_3_O_4_	Optimized polyP encapsulated by SiNPs	Peptide-functionalized gold nanoparticles	Formazan analogs by dithizone and *α*-haloketones reaction	Optimized Remdesivir-loaded L-PLGA NPs	Artemisinin, Artemether, and Artesunate delivery by silver nanoparticles	
**Ligand-receptor binding results**	Interactions with S1-RBD of SARS-CoV-2	Inhibition of binding of ACE2 to S-protein SARS-CoV-2, at a physiological solution	More stable complex with RBD of SARS-CoV-2 than ACE2	Inhibition of SARS-CoV-2 chymotrypsin-like protease, at a physiological solution	Interactions Lisinopril-ACE1 and remdesivir-intracellular targeting protein RdRp	Interactions between negative charges of oxygen atoms of drugs with Ag surface	
**Potential application**	Repurposing medication	Immunologic agents	Antiviral agents	Antiviral agents	Antiviral therapy	Antiviral drugs	

**Table 6:** Nanomaterials based approach to manage SARS-CoV-2.

Nanomaterial	Shape, Size	Strategy	Potential Application	Ref.
Graphene sheets	Layers	Modified graphene sheets conjugated with spike antibody of SARS-CoV-2	Immunodetection	[77]
Gold nanoparticles	2.4 nm Spherical in size	Sulfonated gold nanomaterials	Antiviral agents	[66]
Polymeric nanoparticles	Spherical	Bioinspired DNase-coated melanin-like nanospheres	Sepsis or acute respiratory distress syndrome (ARDS) in sever COVID-19 Patients	[78]
Polymeric nanoparticles	10 nm–1 μm colloidal particles	Ivermectin-delivery by (Poly (lactic-co-glycolic acid)-*b*-Maleimide PEG (polyethylene glycol) Copolymers	Antiviral drug	[79]
Nanostructured lipid carriers	Spherical	Pulmonary delivery of Salinomycin by nanostructured lipid carriers	Drug delivery	[66]
Polymeric nanoparticles	Spherical 22.0 nm	DNase-I-coated polydopamine-PEG poly (ethylene glycol)	Sepsis or acute respiratory distress syndrome (ARDS) in sever COVID-19 Patients	[80]
Decoy nanoparticles	(Not reported)	Fusing genetically engineered cell membrane nanovesicles (293T/ACE2 and THP-1cells)	Therapeutic vaccines	[81]

## 6. Conclusions

As vaccines will not be available for the general population until the end of 2020, approved off-label and experimental drugs that are effective against COVID-19 have been identified, including TMPRSS2 and ACE2 inhibitors, antifungal and antimalarial products, antiviral medicines inhibiting viral RdRp, proteases, virus/host cell membrane fusion inhibitors, and antiviral phytochemicals. In addition to infection preventive strategies that have been implemented in many countries, such as quarantine of infected persons, mobile monitoring of contacts, protection of persons at high risk of infection, restriction of national curfew, and urgent vaccine production and supply, trials of these drugs have been conducted by the WHO and the European Union. This review of therapeutic approaches to COVID-19 aims to facilitate the production of drugs and vaccines against this disease. In future studies, furin inhibitors may be investigated as a strategy for developing SARS-CoV-2 vaccines.

## Figures and Tables

**Table 1:** Virus-based therapy: Drugs capable of attacking nucleotides, nucleosides, or viral nucleic acids of a broad range of coronaviruses and other viruses.

Antiviral Agent	Drug Target	Mechanism of Action	Infectious Disease	References
Remdesivir	RdRp	Terminates the non-obligate chain	SARS-CoV-2, MERS-CoV, SARS-CoV	[20]
Favipiravir	RdRp	Inhibits RdRp	SARS-CoV-2, Influenza	[21]
siRNA	RdRp	Short chains of dsRNA that interfere	SARS-CoV, MERS-CoVWu	[22]
Galidesivir	RdRp	Inhibits viral RNA polymerase function by	Galidesivir SARS-CoV-2,	[23]
Ribavirin	RdRp	Inhibits viral RNA synthesis and mRNA capping	SARS-CoV-2, MERS-CoV, SARS-CoV,	[24]
LJ001 and JL103	Lipid membrane	Membrane-binding photosensitizers that induce	Enveloped viruses (IAV, filoviruses, poxviruses, arenaviruses, bunyaviruses, paramyxoviruses, flaviviruses and HIV-1)	[25]
CR3022	Spike glycoprotein	Immunogenic antigen against Spike protein	SARS-CoV-2, SARS-CoV	[26]
Griffithsin	Spike glycoprotein	Griffithsin binds to the SARSCoV-2 spike	SARS-CoV-2	[27]
Peptide (P9)	Spike glycoprotein	Inhibits spike protein-mediated cell-cell entry or	Broad-spectrum (SARS-CoV, MERS-CoV, influenza)	[28]
Nafamostat	Spike glycoprotein	Inhibits spike-mediated membrane fusion A	SARS-CoV-2, MERS-CoV	[29]
Ritonavir	3CLpro	Inhibits 3CLpro	SARS-CoV-2, MERS-CoV	[30]
Lopinavir	3CLpro	Inhibits 3CLpro	SARS-CoV-2, MERS-CoV, SARS-CoV, HCoV-229E, HIV, HPV	[31]
Darunavir and cobicistat	3CLpro	Inhibits 3CLpro	SARS-CoV-2	[32]

**Table 2:** Host-based therapy: Drug target and mechanism of action against infectious diseases.

Antiviral Agent	Drug Target	Mechanism of Action	Infectious Disease	References
Baricitinib	Clathrin-mediated endocytosis	Baricitinib	Clathrin-mediated endocytosis	[34]
Chloroquine	Endosomal acidification	A lysosomatropic base that appears to disrupt intracellular trafficking and viral fusion events	SARS-CoV-2, SARS-CoV, MERS-CoV	[33]
Convalescent plasma	-	Inhibits virus entry to the target cells	SARS-CoV, SARS-CoV-2, Influenza	[35,36]
Camostat Mesylate	Surface protease	Potent serine protease inhibitor	SARS-CoV, MERS-CoV, HcoV-229E	[33]
Corticosteroids	Pulsed methylprednisolone	Patients with severe MERS who were treated with systemic corticosteroid with or without antivirals and interferons had no favorable response	SARS-CoV, MERS-CoVL	[35]
Nitazoxanide	Interferon response	Induces the host innate immune response	Coronaviruses, SARS-CoV-2	[19]
Recombinant interferons	Interferon response	Exogenous interferons	SARS-CoV-2, SARS-CoV, MERS-CoV	[37]

**Table 3:** Neutralizing antibodies against SARS-CoV-2.

S.N.	Antibody Name	Antibody Type	Origin	PDB ID	Epitopes	Neutralizing Mechanism	Cross Neutralizing Activity	Protective Efficacy	Ref
1	CV30	Human IgG	Infected COVID-19 patients	6XE1	D420-Y421, Y453, L455-N460, Y473-S477, F486-N487, Y489, Q493, T500, G502, Y505	Block hACE2-RBD interaction	no	IC50 value of 0.03 µg/mL	[35]
2	REGN10933 Recombinant	full-human antibodies	Humanized mice and COVID-19-convalescent patients	6XDG	R403, K417, Y421, Y453, L455-F456, A475-G476, E484-Y489, Q493	Block hACE2-RBD interaction, ADCC & ADCP	no	IC50 value of 37.4 pM	
3	B38	Human IgG	COVID-19-convalescent patient	7BZ5	R403, D405-E406, Q409, D420-Y421, Y452, L454-N460, Y473-S477, F486-N487, Y489-F490, Q493-G496, Q498, T500-V503, Y505	Block hACE2-RBD interaction	no	A single dose of B38 (25 mg/kg)	[35]
4	CC12.1	Human IgG	COVID-19-convalescent patient	6XC3	R403, D405-E406, R408-Q409, D420-Y421, Y453, L455-N460, Y473-S477, F486-N487, Y489, Q493-G496, Q498, T500-V503, Y505	Block hACE2-RBD interaction	no	IC50 value of 0.019 µg/mL	[36]
5	CB6	Human IgG	COVID-19-convalescent patient	7C01	R403, D405-E406, R408-Q409, D420-Y421, L455-N460, Y473-S477, F486-N487, Y489, Q493, Y495, N501-G502, G504-Y505	Block hACE2-RBD interaction	no	A single dose of CB6-LALA (50 mg/kg)	[37]
6	C105	Human IgG	COVID-19-convalescent patient	6XCN, 6XCM	R403, D405, R408, D420-Y421, Y453, L455-N460, Y473, A475-G476, F486-N487, G502, Y505	Block hACE2-RBD interaction	no	IC50 value of 26.1 ng/mL	[41]
7	CC12.3	Human IgG	COVID-19-convalescent patient	6XC7	R403, D405, D420-Y421, Y453, L455-N460, Y473-S477, F486-N487, Y489, Q493, G496, N501, Y505	Block hACE2-RBD interaction	no	IC50 value of 0.018 µg/mL	[42]
8	CR3022	Human IgG	SARS-convalescent patient	6YOR, 6 W41	Y369-N370, F374-K386, L390, F392, D428, T430, F515-L517	Trapping RBD in the less stable up conformation while leading to destabilization of S	SARS-CoV, SARS-CoV-2	ND50 value of 0.114 µg/mL	[19]
9	EY6A	Human IgG	Late-stage COVID-19 patient	6ZDH, 6ZER, 6ZCZ	Y369, F374-S375, F377-K386, N388, L390, P412-G413, D427-F429, L517	destabilization of S	SARS-CoV, SARS-CoV-2	ND50 value of ~10.8 µg/mL	[26]
10	VHH-72	Llama single domain antibody	llama immunized with prefusionstabilized betacoronavirus spikes	6WAQ	Y356-T359, F361-C366, A371-T372, G391-D392, R395, N424, I489, Y494	Trapping RBD in the less stable up conformation while leading to destabilization of S, Block hACE2_RBD interaction	SARS-CoV, SARS-Co-V-2	IC50 values of 0.14 µg/mL and 0.2 mg/mL.	[19]
11	BD23	Human IgG	COVID-19-convalescent patient	7BYR	G446, Y449, L452, T470, E484-F486, Y489-F490, L492-S494, G496, Q498, T500-N501, Y505	Block hACE-RBD2 interaction	no	IC50 value of 8.5 µg/mL	[26]
12	Fab 2–4	Human IgG	Infected COVID-19 patients	6XEY	Y449, Y453, L455-F456, E484-F486, Y489-F490, L492-S494, G496	Locking RBD in the down conformation while occluding access to ACE2	no	Neutralizing SARS-CoV-2 live virus with IC50 value of 0.057 µg/mL	[41]

## Data Availability

Data sharing not applicable.
